# A clinical database to assess action levels and tolerances for the ongoing use of Mobius3D

**DOI:** 10.1002/acm2.12009

**Published:** 2016-11-30

**Authors:** David Jolly, Leon Dunn, John Kenny

**Affiliations:** ^1^ Epworth Radiation Oncology Research Centre Epworth HealthCare Melbourne VIC Australia

**Keywords:** action level, database, independent verification, Mobius3D, tolerances

## Abstract

In radiation therapy, calculation of dose within the patient contains inherent uncertainties, inaccuracies, limitations, and the potential for random error. Thus, point dose‐independent verification of such calculations is a well‐established process, with published data to support the setting of both action levels and tolerances. Mobius3D takes this process one step further with a full independent calculation of patient dose and comparisons of clinical parameters such as mean target dose and voxel‐by‐voxel gamma analysis. There is currently no published data to directly inform tolerance levels for such parameters, and therefore this work presents a database of 1000 Mobius3D results to fill this gap. The data are tested for normality using a normal probability plot and found to fit this distribution for three sub groups of data; *Eclipse*,*iPlan* and the treatment site *Lung*. The mean (μ) and standard deviation (σ) of these sub groups is used to set action levels and tolerances at μ ± 2σ and μ ± 3σ, respectively. A global (3%, 3 mm) gamma tolerance is set at 88.5%. The mean target dose tolerance for *Eclipse* data is the narrowest at ± 3%, whilst *iPlan* and *Lung* have a range of −5.0 to 2.2% and −1.8 to 5.0%, respectively. With these limits in place, future results failing the action level or tolerance will fall within the worst 5% and 1% of historical results and an informed decision can be made regarding remedial action prior to treatment.

## 1 Introduction

The delivery of radiation in the oncologic setting plays a vital role in both curative and palliative treatments. The success of such treatments is highly dependent on the accuracy with which they can be delivered to the patient, both geometrically and in terms of physical dose. Furthermore, the preparation of patient delivery is a lengthy process involving a multidisciplinary team and multiple procedures that include medical imaging, patient immobilization, target and organ at risk delineation, dose calculation, image guidance, and the delivery of radiation itself. Each of the above facets have inherent uncertainties and the potential for both random and systematic errors. These must be reduced at each level for the intended clinical treatment to have the highest probability of being successful.

In and of itself, the calculation of patient dose also has multiple levels of potential uncertainty, including but not limited to; computed tomography (CT) to electron density conversion; physical measurement of linear accelerator (linac) beam data, and postprocessing of said data; data transfer; beam modeling/calibration; and lastly patient‐specific factors such as the presence of inhomogeneous media. Generally speaking, radiotherapy centers worldwide will implement a commercial treatment planning system (TPS) from a small number of vendors that employ one of several different methods of dose calculation. Although this commercialization of software has undoubtedly improved consistency across centers, the above factors still exist alongside clinically significant variations, as shown by the results of large‐scale independent auditors^1^, ^2^, ^3^, ^4^, ^5^ and publication of interinstitutional comparisons.^6^ The systems themselves are also highly dependent on user‐defined reference data that is not immune to inadvertent change and/or corruption.

Due to the multifactorial nature and fundamental importance of accurate dose calculation, an independent method of verifying delivery parameters has been recommended by international^7^, ^8^ and national^9^ bodies alike. Historically, these independent methods have been rudimentary in nature — calculating dose to a single point under simplified scatter conditions.^10^, ^11^, ^12^ They are also often based on the same local beam data as the TPS and in practice not fully independent from the original patient‐specific dose calculation, and hence do not give a clinically valuable representation of the uncertainties involved.

An extension to this concept is now commercially available in the form of Mobius3D, independent verification software from (Mobius Medical Systems LP, Houston, TX, USA). In contrast to past monitor unit verification programs, this software utilizes the full patient DICOM set (CT, plan, structure set, and dose) to recalculate 3D dose using a collapsed cone convolution superposition (CCCS) algorithm^13^ and independent reference beam data. The reference beam models were created based on data from multiple machines and further compared to published standards and Imaging and Radiation Oncology Core (IROC)‐Houston consensus data, before being implemented in the Mobius3D system as a golden reference set. This is fundamentally a more rigorous check of the patient dose calculation and, moreover, has the ability to provide information regarding the associated uncertainties of clinical parameters such as mean target dose differences and voxel‐by‐voxel 3D gamma analysis.

Historical point dose verification has analogies with mean target dose in the sense that they converge if point verification is done for all voxels within the target, and 3D gamma comparisons are analogous to physical measurement‐based verification of beam deliveries such as intensity‐modulated radiotherapy (IMRT) and volumetric‐modulated radiotherapy, although these are often limited to planar 2D gamma comparisons. Both of the above analogies are further limited in the sense that they do not include the actual patient geometry in their calculations.

Mobius3D is designed to detect gross errors in treatment planning and therefore the default action levels are inherently loose in nature, but can be further customized by the end user. The authors believe that with appropriate customization, Mobius3D can not only pick up gross errors but inform clinical decision making due to more subtle TPS limitations and uncertainties. Comprehensive commissioning results have been presented previously^14^, ^15^ but neither specifically addressed action levels. The primary aim of the work presented herein is the application of a local clinical database of Mobius3D results, used to inform action levels for these verification parameters. Retrospective statistical analysis is used to tailor action levels based on a number of system parameters (TPS, delivery technique and treatment site). The secondary aim is to not only identify gross errors but to also improve and streamline the treatment planning process in general.

## 2 Materials and methods

### 2.A Materials: treatment planning and delivery systems

As summarized in (Table 1), during the period this work was undertaken, the author's institution had four (Varian Medical Systems, Palo Alto, CA, USA) linacs (LA1‐4) and two multileaf collimator (MLC) models. The treatment planning systems were Varian *Eclipse* V11.0 MR1.0 and BrainLab *iPlan* V4.5.3 (Munich, Germany) with the Anisotropic Analytic Algorithm (AAA) V11.0.31 and Pencil Beam Convolution (PBC) V4.5.3, respectively. Three photon energies were available with 6X‐FFF being exclusively planned in *iPlan* for the stereotactic linacs. Local delivery techniques include static three‐dimensional conformal radiotherapy (3DCRT), sliding window IMRT, dynamic conformal arcs (DCA), and intensity‐modulated radiosurgery (IMRS). The majority of the 3DCRT and IMRT workload was planned in *Eclipse* and delivered on LA1/LA3 (Millennium 120‐MLC), and the DCAs and IMRS planned in *iPlan* and delivered on LA2/LA4 (HD120 MLC), although this assignment of techniques to particular linacs is not exclusive.

**Table 1:** Summary of Epworth radiation oncology system capabilities existing throughout work presented. Note that the checks and crosses for TPS and Delivery Technique indicate generality and not exclusivity

		Linac			
		LA1	LA2	LA3	LA4
Linac type		Trilogy	NovalisTx	21iX	TrueBeam STx
MLC		Millennium 120	HD120	Millennium 120	HD120
Photon energy	6X	✓	✓	✓	✓
	6X–FFF	✗	✓	✗	✓
	10X	✓	✓	✓	✓
TPS	Eclipse	✓	✗	✓	✗
	iPlan	✗	✓	✗	✓
Delivery technique	3DCRT	✓	✗	✓	✗
	IMRT	✓	✗	✓	✗
	DCA	✗	✓	✗	✓
	IMRS	✗	✓	✗	✓

### 2.B Materials: Mobius3D

The Mobius3D server and dose calculation engine comes preconfigured based on a single (per photon energy) user provided percentage depth dose (PDD) value and consensus beam data. There is an ability to customize discrete values of PDD, output factor, and off axis ratio, although locally this was left unmodified as the Mobius3D data were deemed to be sufficiently close to local reference data, and further added to the independent nature of the verification. Adjustments to the Mobius3D dosimetric leaf gap (DLG) offset were made for each permutation of photon energy and MLC model, to align with ion chamber point dose measurements of IMRT plans. Over the period of this work, Mobius3D received several software updates and therefore the data presented include four different versions (V1.5, V1.5.1, V1.5.2, and V1.5.3), although there were no changes to the beam model across these specific versions.

### 2.C Methods: database development and target analytics

The database was formed over a 7‐month period and included the first 1000 clinical calculations. The data were manually extracted and included patient identifying information, date of Mobius3D check, discrete treatment site, delivery technique, linac, photon energy, Mobius3D software version, TPS, primary PTV mean target dose difference (defined in eq. (1) below), and the gamma passing rate (3%, 3 mm). If there was more than one target then the primary PTV was defined as the one receiving the highest mean dose and the target analytics were only recorded for this volume. Analytics for other target structures such as the CTV and/or GTV were also omitted.

(1) $$\%\Delta =\frac{D_{M3D}-D_{TPS}}{D_{Ref}}\times 100\%$$

Where %Δ is the reported mean dose difference, D_M3D_ is the mean target dose as calculated by Mobius3D, D_TPS_ is the corresponding mean target dose as calculated by the local TPS, and D_Ref_ is the maximum point dose in the treatment plan.

The mean (μ) and standard deviation (σ) are calculated for all numeric values recoded, the database as a whole and for subsets of data such as TPS, delivery technique, and certain treatment sites (specifically *lung*). Data are presented as scatter plots over time and histograms showing the distribution of data. The database and TPS subset are tested for normality using a normal probability plot,^16^ a linear regression tool, whereby a normal distribution will lie on a straight line. The purpose of this tool is to assess whether setting action levels based on the standard normal distribution is appropriate and if so then action levels (AL_Δ_) and tolerances (TL_Δ_) will then be set at μ ± 2σ and μ ± 3σ, respectively. If μ has a systematic offset from zero it may be deemed appropriate to set the bounds of these limits asymmetrically around zero and give upper (for example AL_Δ+_) and lower (AL_Δ−_) limits. With this formalism, the TL_Δ_ is defined as the limit of clinical acceptance and the AL_Δ_ at a level set to identify that the result is approaching the TL_Δ_. Assuming a normal distribution this will result in approximately 5% of plans failing the action level and 1% the tolerance, both of which will be crossreferenced to acceptable clinical uncertainty. Finally, the newly developed limits are retrospectively applied to the database to determine the total change in the number of plans breaching the action and tolerance limits.

## 3 Results

**Figure 1:**
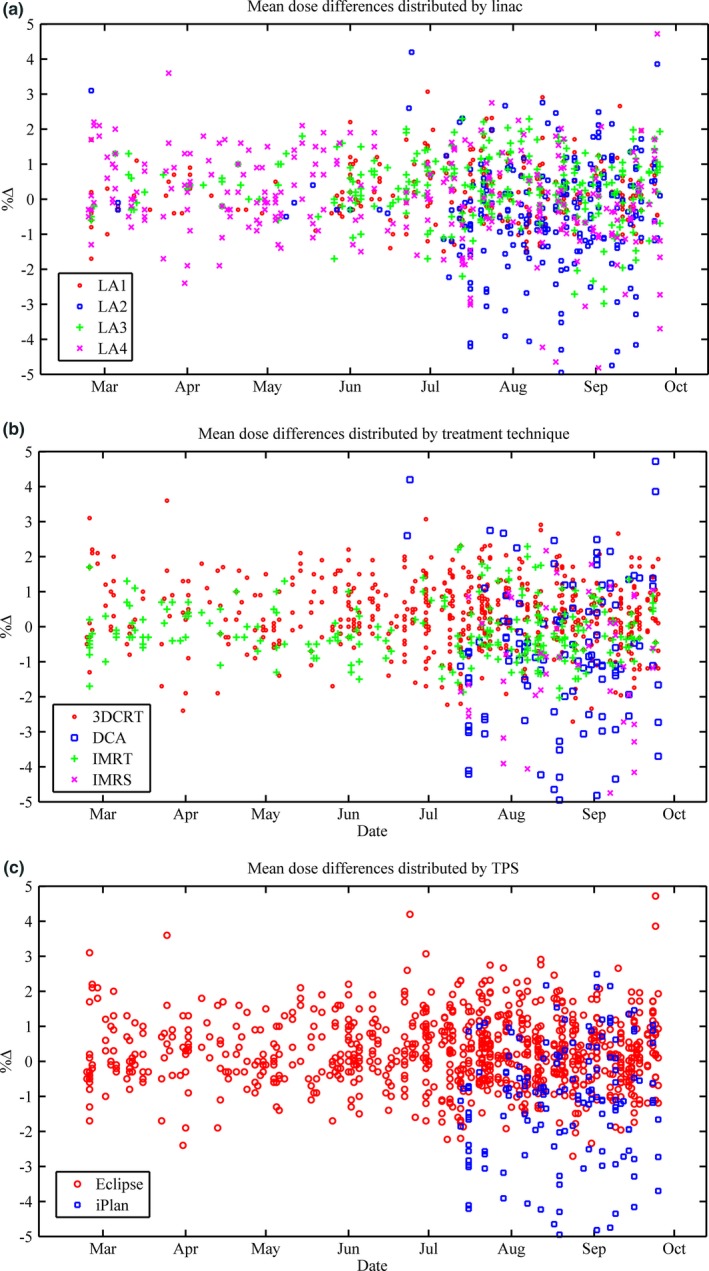
(a‐c): Mean dose difference as defined in eq. (1) over time. Results are broken down into linac (a), delivery technique (b) and TPS (c).

A subset of database results is summarized in (Table 2). Scatter plots of mean dose difference are presented over time (Fig. 1) and further broken down in to linac (1a), delivery technique (1b), and TPS (1c). It is worth noting that the clinical use of M3D saw a somewhat staged roll out starting first with *Eclipse* and then *iPlan*. This affected the total number of patients in any given group, as can most clearly be seen in Fig. 1(c). As previously mentioned, all *iPlan*‐based planning utilizes either DCAs or IMRS and therefore the staging has also affected the numbers in these sub groups. Taken in isolation, Fig. 1(a) may indicate that results are getting worse over time but as can be seen in Fig. 1(b‐c) the majority of these poorer results were DCAs or IMRS planned in *iPlan*. In general, these plans will have much smaller field sizes than *Eclipse* planned 3CDRT/IMRT and are also calculated with a PBC, known to have greater inaccuracies when heterogeneous media are involved.

**Table 2:** Summary of database results. Mean dose difference as defined in eq. (1)

		Number of patients					Mean dose difference (%)				Gamma (3%, 3 mm)			
		Linac					μ	σ	Max	Min	μ	σ	Max	Min
		LA1	LA2	LA3	LA4	Total	−0.06	1.27	4.27	−4.82	97.3	2.9	100.0	79.7
		237	220	272	271	1000								
Technique	3DCRT	146	55	197	197	595	0.35	0.95	3.60	−2.71	96.9	2.5	100.0	82.3
	IMRT	91	42	72	41	246	−0.13	0.81	2.30	−1.87	98.4	1.8	100.0	87.3
	DCA	0	86	0	28	114	−0.70	1.99	4.72	−4.95	97.3	4.2	100.0	79.7
	SRMT	0	37	0	8	45	−0.80	1.68	2.17	−4.75	98.5	1.4	100.0	93.8
Photon energy	6X	60	47	74	84	265	0.47	1.23	4.72	−4.16	97.8	2.5	100.0	86.8
	6FFF	0	113	0	29	142	−1.03	1.64	2.49	−4.95	97.4	3.8	100.0	79.7
	6/10X	40	13	45	18	116	0.45	0.89	2.91	−2.23	97.5	2.5	100.0	86.3
	10X	137	47	150	143	477	0.05	0.85	2.66	−2.40	97.2	2.4	100.0	82.3
TPS	Eclipse	237	106	269	244	856	0.25	0.98	4.72	−2.71	97.4	2.4	100.0	92.3
	iPlan	0	114	0	30	144	−1.10	1.66	2.49	−4.95	97.4	3.8	100.0	79.7
Site	Lung	6	11	3	12	32	1.74	1.18	4.72	0.00	98.2	2.1	100.0	89.7

**Figure 2:**
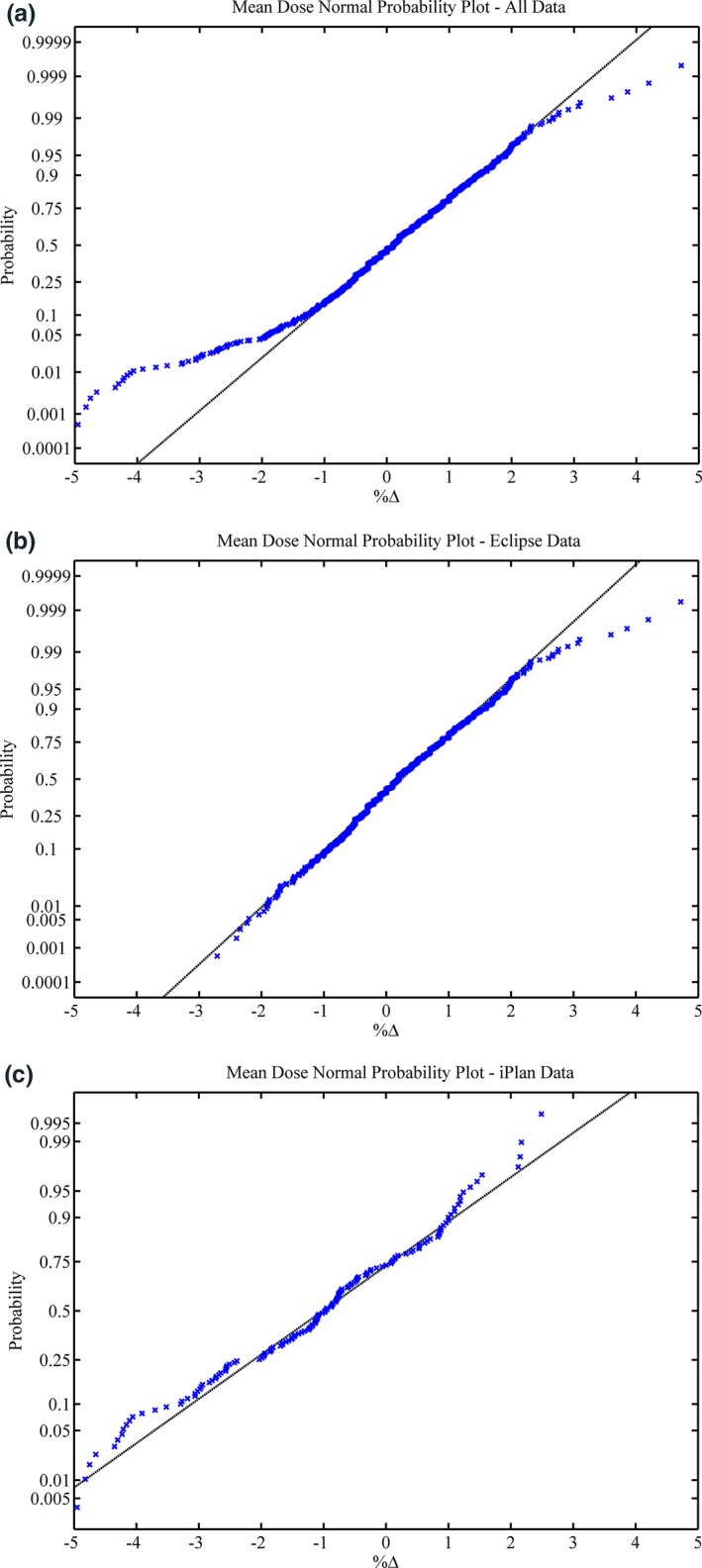
(a‐c): Normal probability plots for all data (a), *Eclipse* (b), and *iPlan* (c).

**Figure 3:**
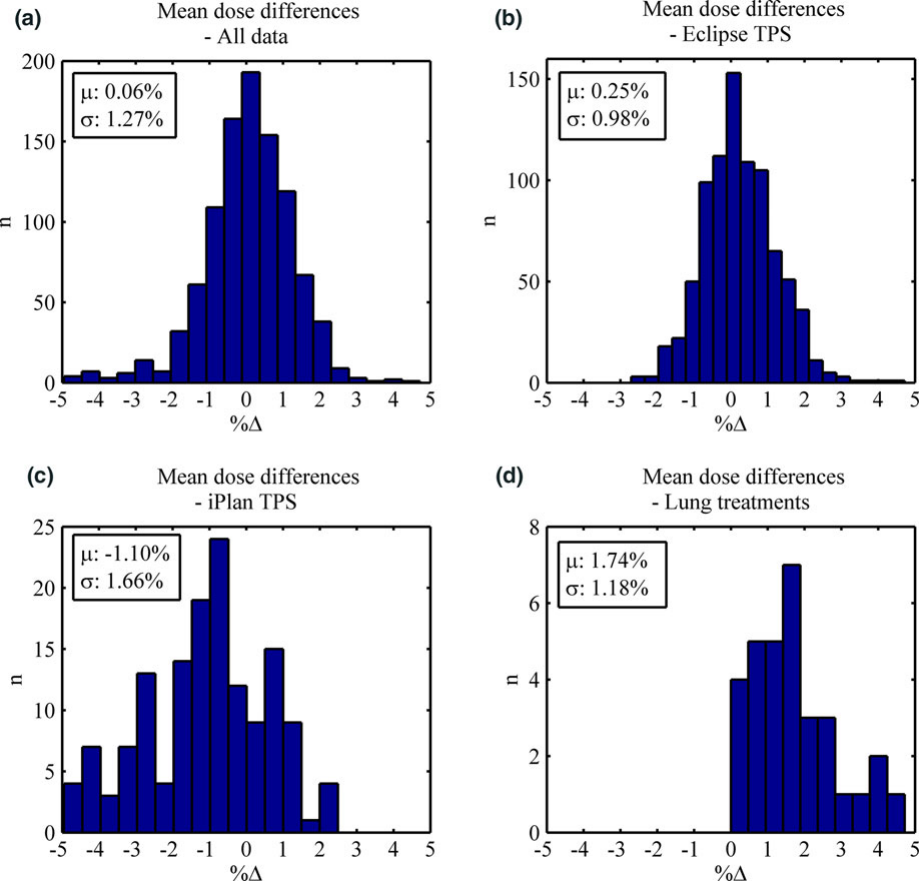
(a‐c): Distributions for the mean dose discrepancies between Mobius and TPS for all data (a), *Eclipse* only (b), *iPlan* only (c), and *Lung* plans. The mean dose and standard deviation of the data are shown inset.

**Figure 4:**
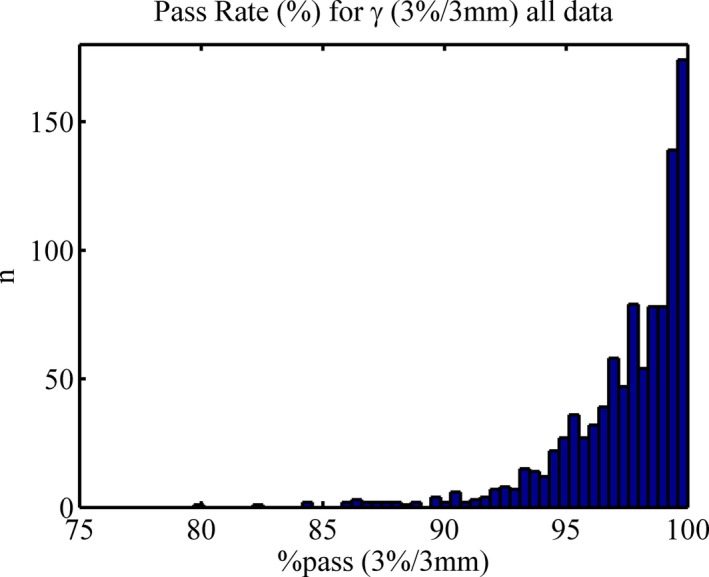
Gamma (3%, 3 mm) histogram for all data.

Normal probability plots are presented in Fig. 2(a‐c) for *All Data*,* Eclipse,* and *iPlan*. Mean dose difference histogram results for *All Data*,* Eclipse*,* iPlan,* and the treatment site *Lung* can be found in Fig. 3(a‐d). All *lung* patients were exclusively planned in *Eclipse* with the AAA, as opposed to *iPlan's* PBC. The gamma results for *All Data* are presented as a histogram in (Fig. 4). The normal probability plot for *All Data* (Fig. 2a) indicates that although the mean dose difference between the TPSs and Mobius3D is almost zero, the data are not normally distributed as a whole and deviates at both the high and low end of the scale. Similar conclusions can be drawn from the mean dose histograms. The *Eclipse* (ignoring *Lung* results deviating at the high end) and *iPlan* data do conform to the linear regression, indicating that the results for these sub groups are normal and thus, the μ and σ can reasonably be used to set tolerance levels. As can be seen in (Fig. 4) the gamma results are skewed due to the nature of the parameter itself, and do not fit a normal distribution. In this case, the application of μ‐ and σ‐based limits were chosen for uniformity and to build in the ability to be variable as more data are added to the database.

Based on these data, the locally defined tolerances and action levels are presented in (Table 3). For simplicity, the small mean dose systematic offset (0.25%) for *Eclipse* has been assumed negligible such that the tolerances can be set symmetrically. For *iPlan* and *Lung*, the systematic differences have been incorporated and therefore the tolerances are nonsymmetric and skewed in the negative and positive direction, respectively. Due to clinical acceptability, instances where calculations exceed 5% (TL_Δ−_ *iPlan* and TL_Δ+_ *Lung*) have been manually capped to this upper limit. The default and proposed levels have been applied to the database and the number of plans failing the specified level is indicated in parentheses. Note here that the default gamma tolerances reference a 5%, 3mm criteria but the database only recorded 3%, 3 mm and thus the true number failing this criteria would likely be much less than 3.3% of the *Eclipse* plans that fail the proposed action level and 0.1% the tolerance, while for *iPlan* these values are 2.8% and 0.7%, respectively.

**Table 3:** Summary of tolerance and action levels. The numerical value in parenthesis indicated the number of plans within the database that fails the specified tolerance or action level

		Mobius3D default	Local		
			Eclipse	iPlan	Lung
Gamma (%)^a^	AL_Δ_	90.0 (20)	91.5 (20)		
	TL_Δ_	85.0 (4)	88.5 (17)		
Mean dose difference (%)	AL_Δ+_	5.0 (0)	2.0 (21)	2.2 (1)	4.1 (2)
	TL_Δ+_	5.0 (0)	3.0 (1)	3.9 (0)	5.0 (0)
	AL_Δ−_	−5.0 (0)	−2.0 (6)	−4.4 (4)	−0.6 (0)
	TL_Δ−_	−5.0 (0)	−3.0 (0)	−5.0 (0)	−1.8 (0)

a Default Mobius3D gamma is based on 5%, 3mm and local is 3%, 3mm.

## 4 Discussion

Independent verification of full 3D dose calculation for every patient is an obvious solution to the complexities involved with patient calculations, but one that has only recently been commercially realized in a meaningful manner. Thus, direct clinical action level and tolerance guidance for these calculations is nonexistent, and comparisons with either clinical acceptability or analogous metrics were the best that one could do. Over approximately 7 months of clinical use, a database of 1000 results has been formed, of which statistical analysis has been used to form local tolerances that could also be used as a baseline for other users worldwide. The caveat here is that these results are based on local TPS (s), beam models, and planning techniques and should therefore only be used as guidance while individual users assess their own results.

The observed differences between TPS and treatment site presented in this work are not unsurprising considering the dose calculation algorithms involved. *Eclipse* utilizes a superposition/convolution type algorithm, *iPlan* a pencil beam convolution, and Mobius3D a collapsed cone. Generally speaking, model‐based calculation algorithms such as the AAA and CCCS have been shown to have a similar level of dose calculation accuracy, whereas PBC has severe limitations, especially when heterogeneous media (as is often found within a real patient) are involved.^17^, ^18^, ^19^

*Lung* treatments tend to involve water‐equivalent targets downstream of relatively low‐density media. In situations like this, the AAA of *Eclipse* has been shown to underpredict dose^5^, ^20^ and CCCS overpredict dose,^21^ both relative to gold standard Monte Carlo calculations. These publications are consistent with local observations whereby Mobius3D systematically calculates more dose to *lung* targets than *Eclipse*, where the reality is most likely to be somewhere in between. In the near future the AcurosXB and Monte Carlo dose calculation engines will be implemented in *Eclipse* and *iPlan,* respectively, and thus new algorithm and treatment site‐specific tolerances will be required.

All 1000 instances over the data acquisition period were within the mean dose difference default value of ±5%, further evidence to the point that tighter values are of clinical benefit. When the newly proposed tolerances were retrospectively applied to the database, the percentage of plans failing action level and tolerance are in line with the specified aims, although the results indicate that a larger number of results may be required for the sub groups *iPlan* and *Lung*. Tolerances tighter than the default levels have been of use throughout the study period to identify various planning metrics that otherwise may have been overlooked. These include but are not limited to: uncertainties in build‐up dose, extreme heterogeneous media such as artificial implants, planning system limitations such as minimum control point spacing for arc‐based treatments (*iPlan* = 10°), dose calculation limitations, and IMRT over modulation, whereby the optimizer had been pushed passed the limit of the associated dose calculation accuracy. Remedial action may have included a replan, change in TPS/calculation algorithm, or even a physical measurement on the linac.

Lastly, it is now possible to access the backend of the Mobius3D database through MATLAB scripting and thus there is potential to automate the presented database and analytic tools. The authors foresee this as a powerful method of simultaneously performing periodic quality assurance on multiple planning systems, verification software, and the planning process in general. This would give clinics the ability to streamline outdated methods and easily track potential change/corruption in many variables over time.

## 5 Conclusion

As with any independent verification method, the question arises as to what action is required when a result exceeds the specified tolerance. By implementation of a clinical database this decision becomes an informed one, due to the fact that the tolerance level is based on a large number of historical results. Following the work presented herein, if a future result(s) falls outside the action level or tolerance, then a good level of confidence can be given to the fact that it is within the worst 5% and 1% of all expected results, respectively. A multidisciplinary approach can then be utilized to make an informed decision regarding the clinical significance and if remedial action is required before treatment proceeds.
